# Inhibition of Virulence-Related Traits in *Pseudomonas syringae* pv. *actinidiae* by Gunpowder Green Tea Extracts

**DOI:** 10.3389/fmicb.2019.02362

**Published:** 2019-10-11

**Authors:** Arianna Lovato, Annalisa Pignatti, Nicola Vitulo, Elodie Vandelle, Annalisa Polverari

**Affiliations:** Biotechnology Department, University of Verona, Verona, Italy

**Keywords:** natural compounds, antimicrobial, kiwifruit, transcriptomics, virulence

## Abstract

Green tea is a widely-consumed healthy drink produced from the leaves of *Camellia sinensis*. It is renowned for its antioxidant and anticarcinogenic properties, but also displays significant antimicrobial activity against numerous human pathogens. Here we analyzed the antimicrobial activity of Gunpowder green tea against *Pseudomonas syringae* pv. *actinidiae* (Psa), the agent that causes kiwifruit bacterial canker. At the phenotypic level, tea extracts strongly inhibited Psa growth and swimming motility, suggesting it could reduce Psa epiphytic survival during plant colonization. The loss of bacterial virulence-related traits following treatment with tea extracts was also investigated by large-scale transcriptome analysis, which confirmed the *in vitro* phenotypes and revealed the induction of adaptive responses in the treated bacteria allowing them to cope with iron deficiency and oxidative stress. Such molecular changes may account for the ability of Gunpowder green tea to protect kiwifruit against Psa infection.

## Introduction

Tea is produced from the leaves of *Camellia sinensis* L. Kuntze and is the most widely-consumed beverage after water. Green tea is a non-fermented tea with a high catechin content, typically consumed in China and Japan but increasingly popular worldwide. It is associated with health benefits such as protection against cancer and cardiovascular disease (Cabrera et al., 2006). The polyphenols in green tea show antimicrobial activity against both Gram-positive and Gram-negative bacteria (Daglia, 2012; Bansal et al., 2013; Reygaert, 2014). The mechanism of action against some human pathogens has been described. For example, tea extracts modulate quorum sensing and reduce the pathogenicity of *Pseudomonas aeruginosa* (Mihalik et al., 2008; Yin et al., 2015). A large body of evidence supports the use of green tea as an inexpensive alternative to conventional antibiotics (Bansal et al., 2013; Liu et al., 2013; Reygaert, 2014; Lade et al., 2015; Yin et al., 2015; Lagha et al., 2017). In contrast, the activity of tea catechins against plant pathogens has not been studied in detail and the mechanism of action is unclear (Yang and Zhang, 2019). There is evidence that tea catechins help to prevent infections by Tobacco mosaic virus and Cucumber mosaic virus (Okada, 1971), as well as the bacterial pathogens *Xanthomonas campestris* pv. *citri* and pv. *vesicatoria*, and *Pseudomonas syringae* pv. *tomato*, which infect citrus and tomato, respectively (Kodama et al., 1991; Lo and Zhao, 2015).

*Pseudomonas syringae* pv. *actinidiae* (Psa) is the causal agent of kiwifruit bacterial canker, the symptoms of which include the brown discoloration of buds, the appearance of necrotic leaf spots surrounded by yellow haloes, cankers on twigs and trunks that yield a reddish exudate, flower and fruit necrosis, and eventually death. The bacterium was first isolated in Japan in 1984 (Takikawa et al., 1989) and subsequently in China (Wang et al., 1992), South Korea, Italy, and New Zealand (Koh et al., 1994; Scortichini, 1994; Chapman et al., 2011; Everett et al., 2011). Between 2008 and 2011, a severe outbreak of the disease caused by a new, genetically distinct strain of the bacterium, appeared in Italy (Ferrante and Scortichini, 2010), and many other growing areas (Cunty et al., 2015). The genetic characterization of different Psa isolates revealed the existence of at least four distinct populations showing different levels of virulence (Ferrante and Scortichini, 2011; Marcelletti et al., 2011), which were subsequently defined as biovars (Vanneste et al., 2013). Biovar 3 is the most virulent and pandemic form, which still causes severe economic losses worldwide (Scortichini et al., 2012). More recently, two other genetically distinct Psa populations have been described: biovar 5 (Fujikawa and Sawada, 2016) and biovar 6 (Sawada et al., 2016). Biovar 4 has been re-classified as a different pathovar, named *actinidifoliorum* (Cunty et al., 2015).

Current integrated control strategies to tackle Psa include the application of copper-based chemicals and preventive measures such as balanced plant nutrition, drip irrigation, the disinfection of pruning shears, the removal of symptomatic plants, and the planting of healthy propagative materials. However, the virulence and rapid spread of the bacterium make the disease difficult to control. Copper-resistant bacterial strains have also been reported (Cha and Cooksey, 1991; Cervantes and Gutierrez-Corona, 1994). Breeding strategies have been developed for the production of resistant cultivars, but only a few wild species with higher Psa tolerance are available as sources of resistance (Nardozza et al., 2015). Several research groups are therefore working to identify effective and environmentally sustainable solutions to control Psa infections, such as increasing plant resistance by applying defense elicitors (Cellini et al., 2014; Wurms et al., 2017), and developing new biocontrol agents (Frampton et al., 2014; Wicaksono et al., 2018) and anti-infective natural compounds (Scortichini, 2014; Song et al., 2016).

Here we investigated the ability of Gunpowder green tea to inhibit Psa growth and virulence-related behaviors. The influence of the tea extracts was also monitored at the transcriptomic level by microarray analysis and real-time RT-PCR. These experiments revealed the ability of the tea to inhibit Psa growth, swimming and biofilm formation *in vitro*, which could reduce epiphytic survival and plant colonization. Moreover, transcriptome analysis revealed the greater abundance of transcripts related to siderophore and biofilm production, aerobic respiration, oxidation-reduction processes and transcriptional regulation. These results suggest the bacterium undergoes transcriptional reprogramming as an adaptive response to iron starvation and oxidative stress, indicating that Gunpowder green tea is a candidate natural anti-infective product for Psa control.

## Materials and Methods

### Green Tea Preparation and the Quantification of Epigallocatechin Gallate

Gunpowder green tea was prepared by incubating 3 g of dried leaves (Peter’s TeaHouse Trading, Bolzano, Italy) in 50 ml of pre-warmed distilled water at 80°C for 30 min (Vuong et al., 2011) followed by filter sterilization (0.2 μm pore size). For each preparation, the quantity of leaf extract was standardized based on the concentration of epigallocatechin gallate (EGCG) measured by UV absorption at 273.5 nm (the EGCG absorption peak) as previously described (Lu et al., 2013). Known concentrations of pure EGCG (Sigma-Aldrich, St. Louis, MO, United States) were used to produce a standard curve for reference.

### Psa Strain and Growth Conditions

The *Pseudomonas syringae* pv. *actinidiae* strain CRAFRU 8.43 (Ferrante and Scortichini, 2010), isolated in 2008 from *Actinidia chinensis* cv. Hort 16A in central Italy (Latina) and kindly provided by Dr. M. Scortichini, was chosen to represent the highly virulent Psa biovar 3. The bacteria were stored at –80°C in 15% (v/v) glycerol and, before each experiment, were freshly streaked onto solid King’s B (KB) agar plates (1.5% w/v agar). Single Psa colonies were transferred to liquid KB medium and incubated at 28°C for 24 h, shaking at 180 rpm, to prepare bacterial cultures for subsequent experiments.

### Antibacterial Assay

The antibacterial activity of Gunpowder green tea was tested on KB agar plates using the paper disk diffusion test (Vincent et al., 1944). Briefly, 100 μl of bacterial suspension (OD_600_ = 1; ∼1 × 10^9^ cells/ml) was uniformly spread over the surface of the plate. Sterile 7-mm disks of Whatman 3MM paper (GE Healthcare Life Sciences, Little Chalfont, United Kingdom) were soaked with 12.5 μl of tea (EGCG content = 15 mg/ml), or 12.5 μl of a 1:2 dilution of the same tea in water (EGCG concentration = 7.5 mg/ml) or sterile water as a negative control. The disks were placed onto the plates and incubated at 28°C for 48 h. We then measured the diameter of growth inhibition zones around the paper disks (longest diameter for oblong haloes). Each experiment was performed in biological triplicates, with three technical replicates per treatment.

### Minimal Inhibitory and Bactericidal Concentrations

The minimal inhibitory concentration (MIC) of the tea was determined using a broth microdilution assay (Stalons and Thornsberry, 1975; Wiegand et al., 2008) in 96-well flat-bottom polystyrene cell culture plates (Sarstedt, Nümbrecht, Germany) containing 200 μl of a Psa cell suspension in liquid KB medium (initial OD_600_ = 0.02). The cells were incubated for 24 h at 28°C in the presence of different quantities of tea corresponding to final EGCG concentrations of 0.4, 0.6, 0.8, and 1 mg/ml, with untreated cells as a positive control. Bacterial growth was monitored by measuring the OD_600_ every hour in a plate reader (BioTek, Winooski, VT, United States). The lowest concentration causing complete growth inhibition was defined as the MIC. Psa viability after treatment was estimated by growing Psa cells (initial OD_600_ = 0.02) for 24 h in liquid KB medium without tea (control) or with different tea concentrations corresponding to 0.4, 0.5, 0.6, and 0.8 mg/ml EGCG. Countable 10-fold dilutions were prepared in liquid KB medium and 100 μl aliquots were spread on KB agar plates. After overnight incubation at 28°C, colonies were counted to determine the number of colony forming units per milliliter (cfu/ml).

The minimal bactericidal concentration (MBC) was assessed by plating out Psa cultures treated with tea at different EGCG concentrations. Briefly, Psa was cultured in liquid KB medium and 200 μl aliquots (initial OD_600_ = 0.02) were transferred to 96-well plates and incubated at 28°C for 24 h with tea at EGCG concentrations ranging from 12 to 0.4 mg/ml. Untreated samples served as positive controls. After incubation, bacterial growth was evaluated by spreading 100 μl of each suspension onto KB solid medium (three different biological replicates for each concentration, each comprising three technical replicates). The plates were incubated for another 48 h at 28°C. The MBC was defined as the lowest EGCG concentration in the liquid medium which devitalized the bacterial cells.

### Siderophore Activity

Psa cells treated with tea were analyzed for siderophore activity using a chrome azurol S (CAS) agar assay (Schwyn and Neilands, 1987). Overnight cultures of Psa grown in KB medium were adjusted to OD_600_ = 0.02, inoculated into KB medium as a control, or into KB medium supplemented with tea corresponding to EGCG concentrations of 0.4 or 0.8 mg/ml, and incubated at 28°C for 24 h, shaking at 180 rpm. Three 12.5 μl droplets of each Psa culture were spotted onto CAS agar plates (Owen and Ackerley, 2011). After incubation at 28°C for 24 h, we measured the diameters of the yellow haloes as well as the longest colony diameters. The size of the yellow halo size depends on efficient transfer of ferric ions from the green/blue CAS complexes to siderophores. The CAS assay was repeated in three independent experiments. The chelating activity of tea was evaluated by spotting 12.5 μl droplets of tea diluted in KB (0.4 or 0.8 mg/ml EGCG) directly onto CAS agar plates, and measuring the resulting yellow haloes after 1 h.

### Lipase Activity

Lipase activity was measured using a tributyrin agar assay (Smeltzer et al., 1992) with some modifications (Patel et al., 2014). We resuspended 5 ml of tributyrin (Sigma-Aldrich, St. Louis, MO, United States) in 25 ml of deionized water using three 60-Hz pulses from a Sonopuls HD2070 tip sonicator (Bandelin Electronic, Berlin, Germany), each lasting 1 min. The tributyrin solution was used to prepare 1% (v/v) tributyrin agar (5 g/L peptone, 3 g/L yeast extract, 15 g/L agar, pH 7.5). Overnight Psa cultures were adjusted to OD_600_ = 0.02, inoculated into KB (control) or KB containing tea (0.4 or 0.8 mg/ml EGCG) and incubated at 28°C for 24 h. Three 12.5 μl droplets of each suspension were then spotted onto tributyrin plates. Psa cells cultured in presence of tea (0.4 or 0.8 mg/ml EGCG) were spotted either onto tributyrin agar (±) or tributyrin agar medium containing the same EGCG concentrations (+/+). Plus (+) and minus (–) symbols were used to specify whether tea was present only during the liquid cultivation (±), only in the agar plates (–/+), or in both (+/+). Untreated Psa cells were also spotted onto tributyrin agar plates without tea (–/–). The activity of extracellular lipases was revealed by the presence of clear zones surrounding the bacterial colonies 7 days after inoculation. We measured the clear zones and the longest colony diameters.

### Swimming Motility Assay

Bacterial motility was measured using a swimming assay (Ha et al., 2014). Psa cells (initial OD_600_ = 0.02) were cultivated for 24 h in liquid KB medium containing tea (0.4 or 0.8 mg/ml EGCG) and three 12.5 μl drops were transferred to KB agar plates overlain with soft KB agar (0.3% w/v agar) containing the same tea content (+/+) or no tea (±). In parallel, Psa cells were cultivated for 24 h in liquid KB medium without tea and were also spotted onto soft KB agar containing tea extracts as above (–/+) or no tea (–/–). Motility was assessed by measuring the longest colony diameter. All the bacterial suspensions were also spotted with the same experimental design on standard KB medium (1.5% w/v agar). Plates were incubated at 28°C for 24 h. Colony diameters on the (–/–) plates were used as a reference value to determine the viability of the Psa cells exposed to tea extracts.

### Assessment of Biofilm Formation

The effect of tea on Psa biofilm formation was determined in 96-well microtiter plates by crystal violet staining (O’Toole and Kolter, 1998). Briefly, overnight cultures of Psa were diluted in KB liquid medium to OD_600_ = 0.5 (2.5 × 10^8^ cells/ml) and incubated in the presence of tea at EGCG concentrations of 0.2, 0.4, 0.6, 0.8, and 1 mg/ml for 72 h under static conditions. Untreated Psa cells cultured in KB medium were used as positive controls, and tea-containing medium without cells was used as a negative control. After the incubation, bacteria were gently removed using a multichannel pipette, and wells were washed three times with distilled water. Psa biofilms were stained with 250 μl 0.1% crystal violet (Sigma-Aldrich, St. Louis, MO, United States) for 20 min. Wells were rinsed again three times with distilled water to remove unbound dye and dried for 1 h at room temperature. The crystal violet dye was solubilized in 250 μl of 30% acetic acid and the biofilm was quantified by measuring the absorbance at 570 nm (OD_570_).

### RNA Isolation, cDNA Synthesis, and Quantitative Real-Time RT-PCR

Psa cells were cultured in liquid KB medium (controls) or in KB medium supplemented with tea (0.4 mg/ml EGCG) for 24 h at 28°C. We then pelleted 2 ml of each bacterial suspension and extracted total RNA from the pellets using the Spectrum Plant Total RNA kit (Sigma-Aldrich, St. Louis, MO, United States). Residual DNA was removed by treating samples with the TURBO DNA-free kit (Thermo Fisher Scientific, Waltham, MA, United States). RNA concentrations were determined using a NanoDrop 2000 spectrophotometer (Thermo Fisher Scientific, Waltham, MA, United States). First-strand cDNA was synthesized from 1 μg total RNA using the SuperScript III Reverse Transcriptase enzyme kit (Invitrogen, Carlsbad, CA, United States). Real-time RT-PCR was performed using the GoTaq qPCR Master kit (Promega, Madison, WI, United States) with a 1:20 dilution of cDNA. Primer sets were designed using the CRAFRU 8.43 reference genome (*Pseudomonas* Genome DB^1^) to detect the transcripts of genes *pvdE* (cyclic peptide transporter; WP_017684002.1), *pvdS* (pyoverdine sidechain peptide synthetase IV; WP_019716667.1), *pvdO* (chromophore maturation protein; WP_017684003.1), *lip_1* (annotated lipase; WP_025987999.1), *aprA* (serine 3-dehydrogenase; WP_017682688.1), *fleQ* (ATPase AAA; WP_003382141.1), *pilM* (pilus assembly protein; WP_003378852.1), *algU* (RNA polymerase sigma factor; WP_003378493.1), *hrpC* (HrcJ family type III secretion inner membrane ring protein; WP_003375729.1), *hrpW1* (type III helper protein; WP_017682518.1) and *gacA* (chemotaxis protein CheY; WP_005737926.1).

To verify the transcriptomic data, six Psa genes that were modulated significantly in the presence of tea were amplified by real-time qPCR: *algD* encoding GDP-mannose 6-dehydrogenase (WP_017683639.1), *fis* encoding a FIS-family transcriptional regulator (WP_002555375.1), *sigma* encoding an RNA polymerase sigma factor (WP_005737468.1), *fliT* encoding a flagellar assembly protein (WP_002554297.1), *fliK* encoding a flagellar hook-length control protein (WP_017683767.1) and the gene for flagellin (WP_003382135.1). Mean normalized expression (MNE) values were based on the constitutive housekeeping gene *rpoD* encoding RNA polymerase sigma factor WP_017683803.1. All primer sets used for real-time RT-PCR were first tested by conventional PCR (Supplementary Table S1). Real-time RT-PCR was carried out using the StepOnePlus Real-Time PCR System (Applied Biosystems, Foster City, CA, United States) under the following conditions: initial heating to 95°C for 2 min followed by 40 cycles of 95°C for 15 s, 55°C for 30 s and 60°C for 30 s, followed by melting curve analysis to check primer specificity. The comparative threshold cycle method (ΔΔCt) was used for the analysis of transcript levels. Results are presented as MNE values relative to *rpoD*. Mean and standard error (SE) values were determined from three biological replicates.

### Microarray and Data Analysis

The Psa transcriptome was interrogated using a custom SurePrint G3 GE 8 × 60K chip designed in house and produced by Agilent Technologies (Santa Clara, CA, United States). CRAFRU 8.43 gene features and annotations were retrieved from GenBank Assembly GCA_000233815.2 (ASM23381v2). The input sequence collection representing the whole CRAFRU 8.43 genome and encompassing the nucleotide sequences of 7919 coding regions (CDSs) was provided to the Agilent eArray web tool^2^ to facilitate probe design (Colombo, Vandelle, Polverari, unpublished results; GEO platform ID: GPL27505)^3^. The results of the hybridizations were analyzed using Agilent G4900DA SureScan Microarray Scanner System with the Agilent Scan Control software and the data were extrapolated using the Agilent Feature Extraction software (2010). The raw data obtained were normalized, statistically evaluated and processed. Briefly, we calculated the average and the standard deviation value of the triplicates-probe present in the microarray, then the data were normalized using the non-parametric tests. Differentially expressed genes were filtered considering a false discovery rate (FDR) of 0.1 and log_2_ FC > |0.5|. Enrichment analysis bar charts and word clouds were produced using the Blast2GO platform (Conesa and Götz, 2008).

### Statistical Analysis

All *in vitro* experiments were performed as three independent replicates. Means, standard deviations (SD) and standard errors (SE) were calculated and significance was determined using Student’s *t*-test. All results were considered statistically significant at *p* < 0.05 (^∗^) and *p* < 0.01 (^∗∗^).

### Efficacy Tests in Kiwifruit Plants

Experiments were carried out in a growth chamber under controlled conditions (20°C and 60% relative humidity). Two-month-old kiwifruit plants (*Actinidia deliciosa* cv. Hayward) grown in pots until the three-leaf stage, were evenly sprayed until dripping with tea at a concentration corresponding to half-MBC (4.7 mg/ml EGCG) or with water as a negative control, using a manual pressure nebulizer. We sprayed 25 plants in each treatment group. Plants were artificially infected 1 day after treatment by spraying all the expanded leaves at high pressure with a Psa suspension of 10^7^ cells/ml (OD_600_ = 0.02) in 10 mM MgCl_2_ so that a standardized infection could be induced, reaching 100% infection in the plants that were not sprayed with tea prior to Psa exposure. Plants were checked daily for 14 days to identify disease symptoms (necrotic spots) on the leaves emerging from the apex after infection. Symptom severity was scored on these apical expanding leaves (not directly sprayed) as follows: 0 spots = no symptoms; 1–10 spots = mild symptoms; > 10 spots = severe symptoms. Data were presented as the percentage of plants in each treatment group showing different scores at 7 and 14 days post-infection (dpi).

## Results

### Gunpowder Green Tea Inhibits Psa Growth and Viability

**FIGURE 1:**
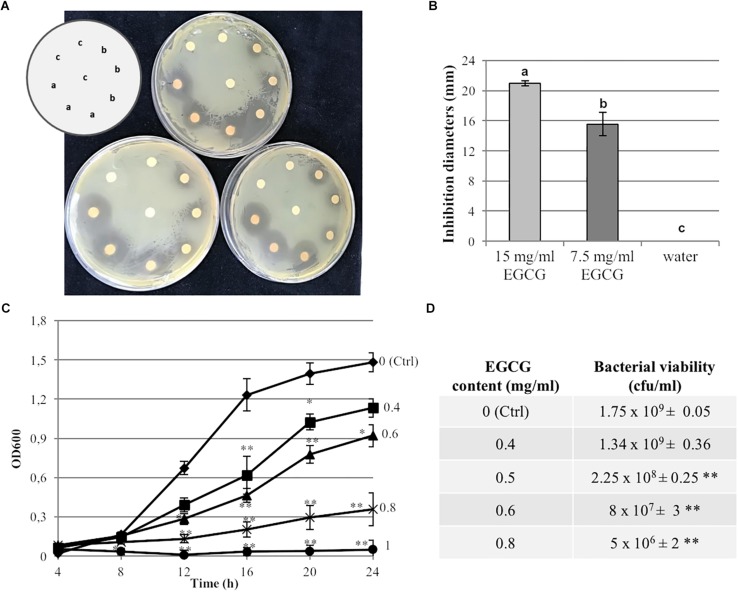
Antibacterial activity of Gunpowder green tea against *P. syringae* pv. *actinidiae*. **(A)** Growth of *Pseudomonas syringae* pv. *actinidiae* CRAFRU 8.43 (Psa) in the presence of paper disks spotted with two different concentrations of tea: (a) undiluted (EGCG 15 mg/ml) or (b) diluted 1:1 with water (EGCG 7.5 mg/ml). Water-spotted discs were used as negative controls (c). Pictures were taken after 48 h. **(B)** Measurement of halo diameters under the conditions described in **(A)**. The values are expressed as means ± SD of three biological replicates. Different letters indicate statistically significant differences among samples according to ANOVA and Tukey’s HSD. **(C)** Antibacterial activity of tea against Psa grown in microtiter plates for 24 h in presence of diluted tea extracts corresponding to different final EGCG concentrations (0.4, 0.6, 0.8, and 1 mg/ml). The OD_600_ was measured at different time points as indicated and compared to the control (Ctrl, no tea). **(D)** Viable counts (cfu/ml) of Psa grown on solid KB medium for 24 h in the presence of diluted tea extracts corresponding to different final EGCG concentrations (0.4, 0.5, 0.6, 0.8 mg/ml) compared to the control (Ctrl, no tea). Values are expressed as means ± SD of three biological replicates. Asterisks indicate a statistically significant difference (^∗^*p* < 0.5; ^∗∗^*p* < 0.01) according to Student’s *t*-test.

Undiluted Gunpowder green tea (15 mg/ml EGCG) and a diluted extract (7.5 mg/ml EGCG) inhibited Psa growth in a paper disk diffusion assay (Figure 1A). Significant haloes indicating antibacterial activity were observed at both concentrations, with an average diameter of 21 mm at the highest tea concentration and 15.5 mm with the diluted tea (Figure 1B). In contrast, no halo was observed in the negative controls treated with water. Psa cultivated for 24 h in liquid KB medium supplemented with different concentrations of tea (corresponding to 0.4, 0.6, 0.8, and 1 mg/ml EGCG) showed significant growth inhibition compared to the control with no tea (Figure 1C). The MIC was 1 mg/ml EGCG. To determine whether the lower OD_600_ was due to bactericidal or bacteriostatic effects, the same Psa cultures grown overnight in the presence of different concentrations of tea were plated to determine the cfu/ml (Figure 1D). This revealed that the presence of tea at an EGCG concentration of 0.8 mg/ml significantly inhibited bacterial growth kinetics but did not kill the bacteria, which were still viable at the end of the experiment, with approximately the same cell number as the initial inoculum. In contrast, with an EGCG concentration of 0.4 mg/ml, the tea significantly affected the bacterial growth kinetics only at the earlier time points, whereas the number of viable bacteria (cfu/ml) did not differ significantly from the untreated control after incubation for 24 h. Finally, the MBC was estimated over a broad range of tea dilutions, revealing that tea with an EGCG concentration ≥ 9.5 mg/ml completely killed Psa after incubation for 24 h.

### Gunpowder Green Tea Inhibits Psa Iron-Chelating Activity

**FIGURE 2:**
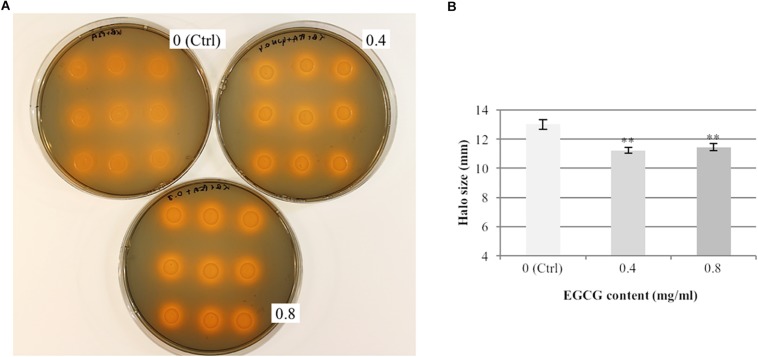
Gunpowder green tea reduces the siderophore activity of *P. syringae* pv. *actinidiae*. *P. syringae* pv. *actinidiae* (Psa) cells were cultivated for 24 h in KB medium (Ctrl) or KB medium supplemented with tea (0.4 and 0.8 mg/ml EGCG), spotted 12.5 μl of the culture onto CAS agar plates and incubated them for 24 h at 28°C. **(A)** Representative plates showing the orange haloes due to the chromazurol indicator around the colonies, indicating iron sequestration by siderophores. **(B)** Halo sizes were measured after 24 h. Values are expressed as means ± SD of triplicate assays from three independent experiments. Asterisks indicate a statistically significant difference (^∗∗^*p* < 0.01) according to Student’s t-test.

To test the hypothesis that Gunpowder green tea inhibits bacterial virulence-related phenotypes, we measured Psa siderophore activity using a CAS assay. Tea (0.4 and 0.8 mg/ml EGCG) slightly inhibited bacterial siderophore activity in liquid medium compared to the control (Figures 2A,B). Notably, neither of these tea concentrations affected bacterial growth on solid KB medium, resulting in colonies ∼8 mm in diameter both in the treated and untreated samples (Supplementary Figure S1A).

Given that tea polyphenols (including EGCG) are thought to possess iron-chelating activity (Hider et al., 2001; Perron and Brumaghim, 2009; Lagha et al., 2017), we determined whether tea alone, at the same concentrations described above, could produce haloes in the absence of Psa (Supplementary Figure S1B). After 1 h, the average halo sizes were 9.2 and 9.7 mm for EGCG concentrations of 0.4 and 0.8 mg/ml, respectively (Supplementary Figure S1C). The orange haloes produced by Psa in the presence of tea (Figure 2B) after 24 h on CAS plates were thus only slightly larger (15–18%) than those produced by tea alone after 1 h (Supplementary Figure S1C). These results indicate that Gunpowder green tea significantly reduces Psa siderophore activity, but the activity is likely to be underestimated due to the strong chelating effect of the tea itself.

### Gunpowder Green Tea Slightly Inhibits Psa Lipase Activity

**FIGURE 3:**
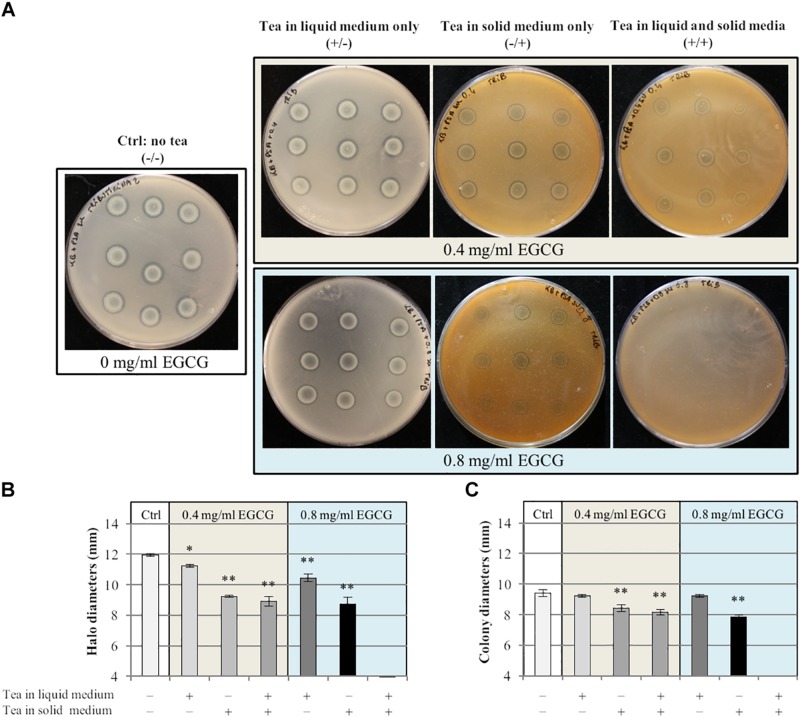
Gunpowder green tea reduces the lipase activity of *P. syringae* pv. *actinidiae*. **(A)** Representative plates showing *P. syringae* pv. *actinidiae* (Psa) lipase activity under different conditions and the corresponding halo sizes **(B)** and colony diameters **(C)**. Psa cells grown for 24 h in KB medium with or without tea (0.4 or 0.8 mg/ml EGCG) were spotted onto tributyrin agar plates with or without tea (0.4 or 0.8 mg/ml EGCG) as indicated. Plates were incubated for 7 days at 28°C. Clear haloes around growing colonies indicated lipase activity. Values are expressed as means ± SD from three independent assays. Asterisks indicate a statistically significant difference (^∗∗^*p* < 0.01; ^∗^*p* < 0.05) according to Student’s *t*-test.

The activity of Psa secreted lipases was evaluated using a tributyrin agar assay (Figure 3). As expected, lipase activity led to the formation of a transparent halo around the Psa colonies 7 days after inoculation (Figure 3A). However, in the presence of tea the halo sizes decreased in a dose-dependent manner compared to the (–/–) untreated control (Figures 3A,B). In particular, a strong reduction of lipase activity (12% decrease of halo diameter) was detected when Psa cells were cultured overnight in the presence of tea (0.8 mg/ml EGCG) and spotted on tea-free tributyrin agar plates (±). There was a smaller reduction (6% decrease of halo diameter) in the presence of diluted tea (0.4 mg/ml EGCG). Importantly, the loss of lipase activity did not correlate with a smaller colony diameter, confirming that the tea affected Psa lipase activity directly rather than by suppressing bacterial growth. Conversely, when the tea was also added to the tributyrin agar plates (−/+, +/+), the loss of lipase activity was strongly associated with Psa growth inhibition (Figure 3C).

### Gunpowder Green Tea Reduces Psa Motility and Biofilm Formation at Sub-MIC Concentrations

**FIGURE 4:**
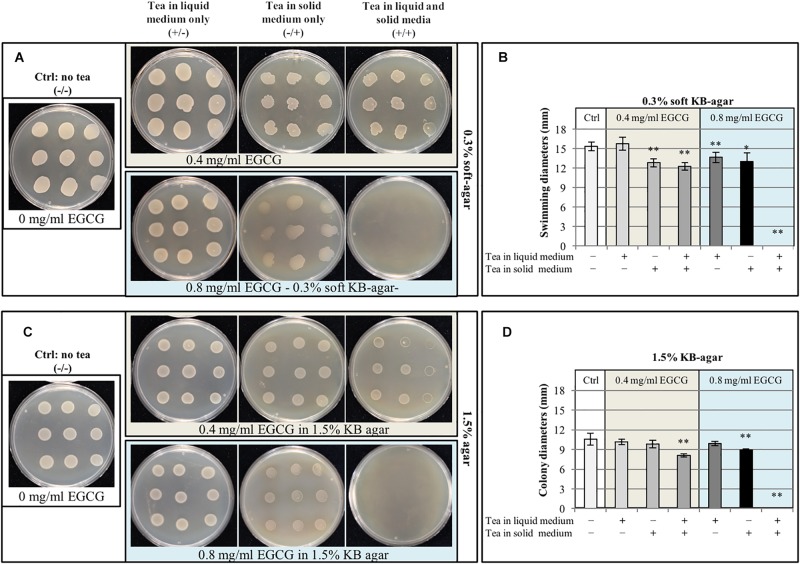
Gunpowder green tea impairs *P. syringae* pv. *actinidiae* motility. **(A,B)** *P. syringae* pv. *actinidiae* (Psa) cell suspensions grown in liquid KB medium with or without tea (0.4 or 0.8 mg/ml EGCG) were spotted onto 0.3% soft KB agar with or without tea (0.4 or 0.8 mg/ml EGCG) as indicated and colony diameters were measured to determine swimming capacity. Pictures of representative plates **(A)** and colony diameter measurements **(B)** were taken after incubation for 24 h at 28°C. **(C,D)** The same bacterial suspensions as above (with or without tea, 0.4 or 0.8 mg/ml EGCG) were also spotted onto standard KB agar plates (1.5% agar) with or without tea (0.4 or 0.8 mg/ml EGCG) as indicated, to evaluate bacterial vitality. Pictures of representative plates **(C)** and colony diameter measurements **(D)** were taken after incubation for 24 h at 28°C. Values are expressed as means ± SD from three independent assays. Asterisks indicate a statistically significant difference (^∗∗^*p* < 0.01; ^∗^*p* < 0.05) according to Student’s *t*-test.

The swimming capacity of Psa was evaluated by inoculating overnight cultures grown in the presence or absence of tea (0.4 or 0.8 mg/ml EGCG) on soft KB agar with or without tea at the same concentrations, giving four treatment combinations at each concentration of EGCG (Figure 4). We found that Psa swimming capacity was significantly reduced in almost all samples containing tea before and/or after plating compared to the (–/–) control with the exception of cultures cultivated in the presence of 0.4 mg/ml EGCG and spotted on soft KB agar without tea (±). Interestingly, as well as influencing bacterial motility, the tea also affected the shape of the Psa colonies, with the appearance of dendrites particularly in plates supplemented with the extract (−/+, +/+). This may be a consequence of the modification of motility.

To evaluate whether the inhibition of swimming was associated with the loss of bacterial viability, all bacterial suspensions were also spotted onto KB containing 1.5% agar for a standard vitality assay. The presence of tea also affected bacterial growth, but only when both the liquid culture and soft agar plates were supplemented (+/+) or when tea was solely present in the soft agar at the highest concentration of 0.8 mg/ml EGCG (Figures 4C,D). As stated above, the presence of tea at the highest EGCG concentration of 0.8 mg/ml in both the liquid cultures and agar completely inhibited Psa growth (regardless of the agar concentration). These data show that the high concentration of Gunpowder tea extract reduces Psa growth and motility, whereas the lowest concentration has a significant effect on swimming motility without inhibiting growth.

**FIGURE 5:**
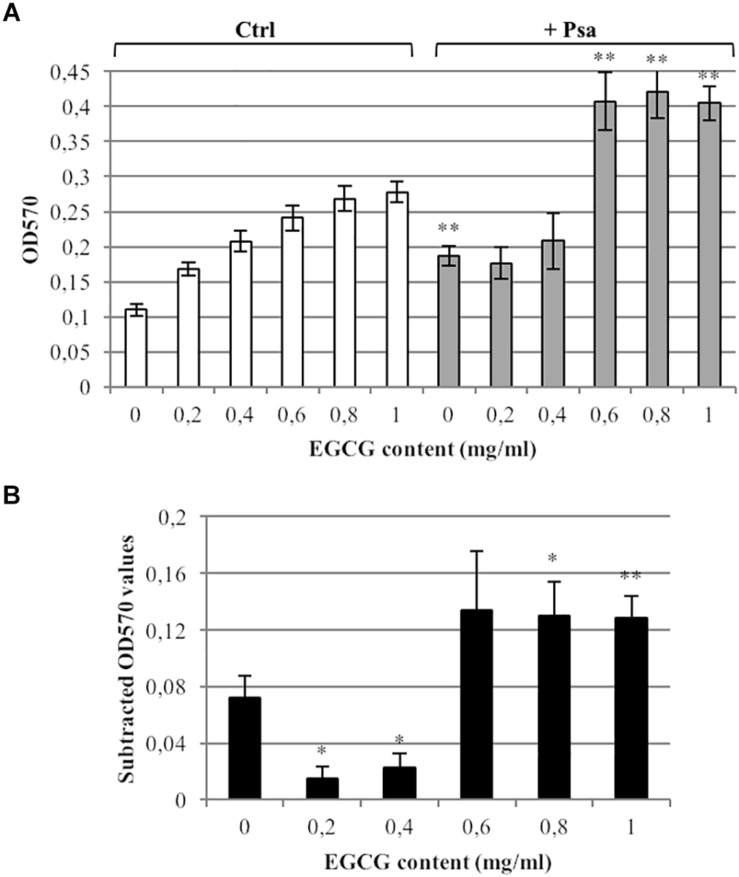
Gunpowder green tea affects *P. syringae* pv. *actinidiae* biofilm formation. **(A)** Biofilm quantification in *P. syringae* pv. *actinidiae* (Psa) cell suspensions cultured in 96-well plates for 72 h at 28°C in KB medium (no tea) or KB medium supplemented with diluted tea extracts (0.2, 0.4, 0.6, 0.8, and 1 mg/ml EGCG; gray bars) stained with crystal violet. Absorbance was measured at OD_570_. Wells containing no cells, with or without tea (Ctrl, white bars) were used as negative controls. Asterisks indicate a statistically significant difference compared to the corresponding controls (^∗∗^*p* < 0.01; ^∗^*p* < 0.05) according to a paired Student’s *t*-test. **(B)** For clarity, the absorbance value of each control was subtracted from the absorbance value of each corresponding Psa sample. The measurements of crystal violet staining were performed after incubation for 72 h. Values are expressed as means ± SE of three technical repeats from three independent experiments. Asterisks indicate a statistically significant difference between the “no tea” (0 mg/ml) value and the values for different EGCG concentrations (^∗∗^*p* < 0.01; ^∗^*p* < 0.05) according to a paired Student’s *t*-test.

The analysis of biofilm formation by crystal violet staining showed that Psa strain CRAFRU 8.43 is a poor biofilm producer, at least under our conditions (Figure 5; EGCG content 0 mg/ml). Moreover, it is worth noting that the green tea extract itself is responsible for crystal violet staining, according to the absorbance increase observed in control samples with different concentrations of extract, in a dose-dependent manner (Figure 5A; white bars). For that reason, the actual effect of tea extract on biofilm production was calculated by subtracting control values from values obtained with Psa-containing samples (Figure 5B). This revealed that the addition of tea extract to the medium completely abolished biofilm formation at 0.2 or 0.4 mg/ml EGCG (Figure 5B). Surprisingly, we observed a statistically significant increase in biofilm formation in the presence of tea extracts with EGCG concentrations exceeding 0.8 mg/ml after incubation for 3 days (Figure 5B).

### Gunpowder Green Tea Modulates the Expression of Genes Related to Psa Virulence

**FIGURE 6:**
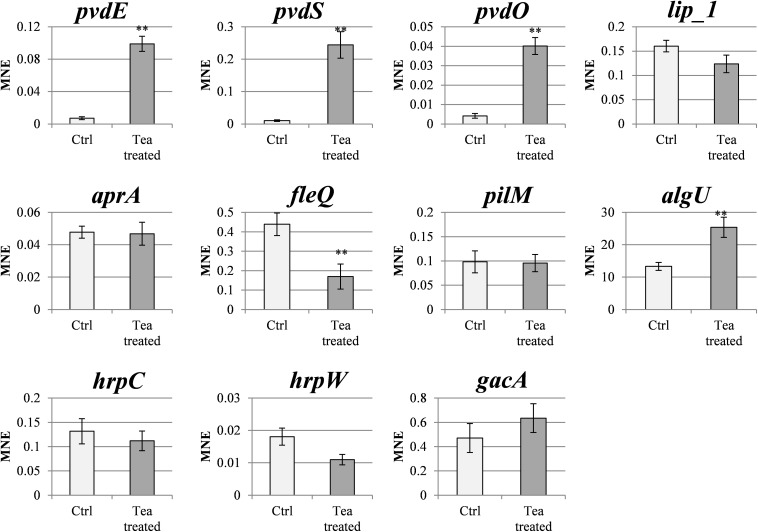
Gunpowder green tea modulates the expression of *P. syringae* pv. *actinidiae* virulence-related genes. Expression profiles of *pvdE*, *pvdS*, *pvdO*, *lip_1*, *aprA*, *fleQ*, *pilM*, *hprC*, *hrpW*, and *gacA* genes in tea-treated *P. syringae* pv. *actinidiae* (Psa) cells (cultured for 24 h in the presence of tea, 0.4 mg/ml EGCG) compared to untreated (Ctrl) cells cultured in KB medium without tea. The expression levels were determined by qRT-PCR and normalized to *rpoD* expression. Data are expressed as mean expression values (MNEs) ± SD of three independent experiments. Asterisks indicate a statistically significant difference (^∗∗^*p* < 0.01) according to Student’s *t*-test (*pvdE*, cyclic peptide transporter; *pvdS*, pyoverdine sidechain peptide synthetase IV; *pvdO*, chromophore maturation protein; *lip_1*, annotated lipase; *aprA*, serine 3-dehydrogenase; *fleQ*, ATPase AAA; *pilM*, pilus assembly protein; *hrpC*, *hrcJ* family type III secretion inner membrane ring protein; *hrpW*, type III helper protein; *gacA*, chemotaxis protein cheY; *rpoD*, RNA polymerase sigma factor).

The effect of sub-MIC doses of tea (0.4 mg/ml EGCG) on Psa was investigated at the molecular level by analyzing the expression of selected genes representing bacterial virulence-related pathways by RT-qPCR (Figure 6). Psa genes related to siderophore production (*pvdE*, *pvdS*, and *pvdO*) were expressed at a low basal level but were strongly upregulated (10–20-fold) in the presence of tea. Interestingly, *fleQ* (which regulates flagellar biogenesis and coordinates flagella-dependent and flagella-independent motility) was downregulated 2.6-fold in the presence of tea, correlating with the reduced swimming motility described above. However, we observed no modulation of *pilM*, which encodes a protein required for pilus assembly and pilus-dependent motility (twitching). The *algU* gene, which regulates alginate biosynthesis and biofilm formation, was upregulated twofold in the presence of tea. Conversely, *lip_1* and *aprA* (encoding a lipase and protease, respectively) were not modulated. Finally, no significant changes were detected in the expression of genes encoding type III secretion system proteins (*hrpC* and *hrpW*) or the master regulator *gacA*.

### Microarray Analysis Reveals That Gunpowder Green Tea Affects Diverse Biological Processes in Psa

**FIGURE 7:**
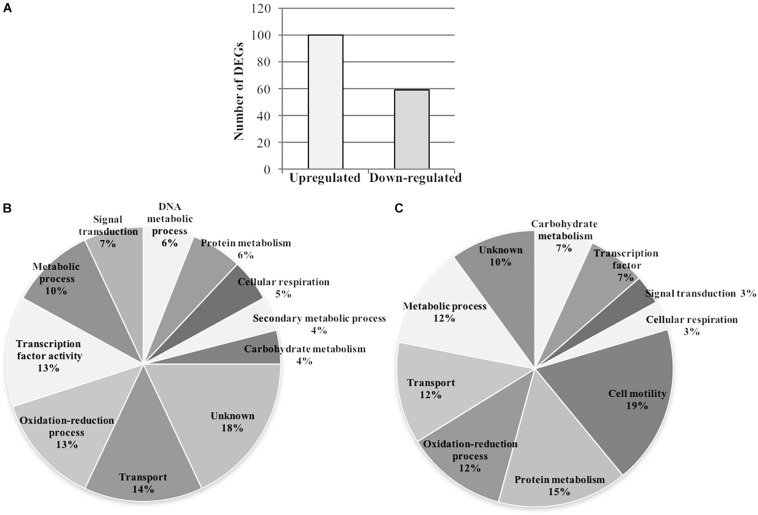
Gunpowder green tea treatment modulates the transcriptome of *P. syringae* pv. *actinidiae*. *P. syringae* pv. *actinidiae* (Psa) cell suspensions were treated with tea (0.4 mg/ml EGCG) for 24 h and harvested for RNA extraction and microarray analysis. **(A)** Total number of upregulated and downregulated Psa transcripts, following treatment with tea. **(B,C)** Distribution of the functional categories of Psa transcripts upregulated **(B)** or downregulated **(C)** by tea. DEGs, differentially expressed genes.

Based on the targeted analysis of virulence-related genes, we investigated the broad transcriptomic responses of Psa in the presence of tea to gain deeper insights into the corresponding molecular pathways. We therefore treated Psa with the same sub-MIC dose of tea (0.4 mg/ml EGCG) and hybridized cRNA prepared from the treated cells to a microarray containing the complete genome of Psa strain CRAFRU 8.43. Statistical analysis (FDR < 0.1 and log_2_FC > | 0.5|) revealed a set of 159 differentially expressed genes, 100 of which were upregulated and 59 downregulated (Figure 7A and Supplementary Table S2). The microarray data were confirmed by real-time qPCR analysis of a subset of these genes, which confirmed the induction of *algD*, *fis* and *sigma* (encoding a sigma factor) in the presence of tea, and likewise the downregulation of *fliT* and *flagellin*. In contrast, the modulation of *fliK* was not confirmed by microarray analysis (Supplementary Figure S2 and Supplementary Table S2). The upregulated group included many genes related to carbohydrate biosynthesis, biofilm production and transcription factors involved in different bacterial responses, whereas the downregulated group included numerous genes involved in flagellin metabolism (Table 1).

**TABLE 1:** Differentially expressed genes belonging to major functional categories modulated by tea treatment in *P. syringae* pv. *actinidiae*.

**Protein ID**	**Log_2_ FC**	**Adjusted *p*-value**	**Gene product description**
**Motility**			
WP_003382135.1	–1.37	0.00733	Flagellin
WP_003375857.1	–1.08	0.01946	Chemotaxis protein (*cheW*)
WP_017683767.1	–0.96	0.01692	Flagellar hook-length control protein (*fliK*)
WP_002554297.1	–0.84	0.01734	Flagellar assembly protein (*fliT*)
WP_003382137.1	–0.82	0.04297	Flagellar protein (*flaG*)
WP_003382140.1	–0.82	0.03051	Flagellar biosynthesis protein (*fliS*)
WP_019716561.1	–0.77	0.03117	Flagellar hook protein (*fliD*)
WP_002554316.1	–0.70	0.01851	Flagellar basal body rod protein (*flgC*)
WP_005616790.1	–0.65	0.06866	Flagellar basal body rod protein (*flgF*)
WP_003382142.1	–0.65	0.05024	Flagellar sensor histidine kinase
WP_020315304.1	–0.65	0.05803	Type IV pilin
WP_017682904.1	–0.52	0.03554	Chemotaxis protein
WP_003382124.1	–0.52	0.09871	Flagellar basal body rod protein (*flgG*)
**Carbohydrate biosynthesis**			
WP_003382270.1	–0.69	0.02302	glucose-6-phosphate isomerase
WP_017683513.1	–0.59	0.062817	Xylulokinase
WP_005737846.1	–0.51	0.068046	Glycogen operon
WP_017683843.1	0.53	0.06281	UDP-N-acetylglucosamine 2-epimerase
WP_024533197.1	0.57	0.07825	Beta-glucosidase
WP_017683184.1	0.61	0.03554	Sigma factor regulatory protein (*algU*)
WP_017683639.1	0.68	0.01691	GDP-mannose 6-dehydrogenase (*algD*)
WP_017683184.1	0.76	0.02302	Sigma factor regulatory protein (*algU*)
WP_003376143.1	0.82	0.04565	Polysaccharide deacetylase family
**Transcription factor activity**			
WP_004396282.1	–1.23	0.00199	Transcriptional regulator (*merR*)
WP_017683822.1	–0.96	0.01850	Transcriptional regulator (*luxR*)
WP_003380805.1	–0.66	0.02714	Family transcriptional regulator (*gntR*)
WP_003382520.1	0.53	0.06308	Chemotaxis protein (*cheY*)
WP_003380601.1	0.54	0.04725	Fe-S assembly transcriptional regulator (*iscR*)
WP_002555375.1	0.57	0.09333	Fis family transcriptional regulator
WP_003377326.1	0.61	0.033040	Transcriptional regulator
WP_003382520.1	0.61	0.03153	Chemotaxis protein (*cheY*)
WP_003378621.1	0.69	0.07232	Transcriptional regulator (*luxR1*)
WP_003380559.1	0.70	0.02362	RNA polymerase sigma 70
WP_003382180.1	0.76	0.02053	RNA polymerase sigma factor
WP_002555375.1	0.78	0.02714	Transcriptional regulator (*fis*)
WP_003382992.1	0.84	0.04615	RNA polymerase sigma 70
WP_005737468.1	0.88	0.01051	RNA polymerase sigma factor (*sigma*)
WP_003379782.1	1.36	0.00068	Transcriptional regulator

Figure 7B shows the functional annotation of the upregulated transcripts based on Gene Ontology (GO) categories, revealing genes mainly involved in transport (14%), oxidation-reduction processes (13%), transcription factor activity (13%) and metabolic processes (10%), together with less common categories such as signal transduction (7%), DNA metabolic processes (6%), protein metabolism (6%), cellular respiration (5%), secondary metabolic processes (4%), and carbohydrate metabolism (4%). Figure 7C shows the corresponding annotation of the downregulated transcripts, revealing prominent categories such as cell motility (19%), protein metabolism (15%), oxidation-reduction processes (12%), transport (12%) and metabolic processes (12%), together with less common categories such as carbohydrate metabolism (7%), transcription factor activity (7%), signal transduction (3%), and cellular respiration (3%).

**FIGURE 8:**
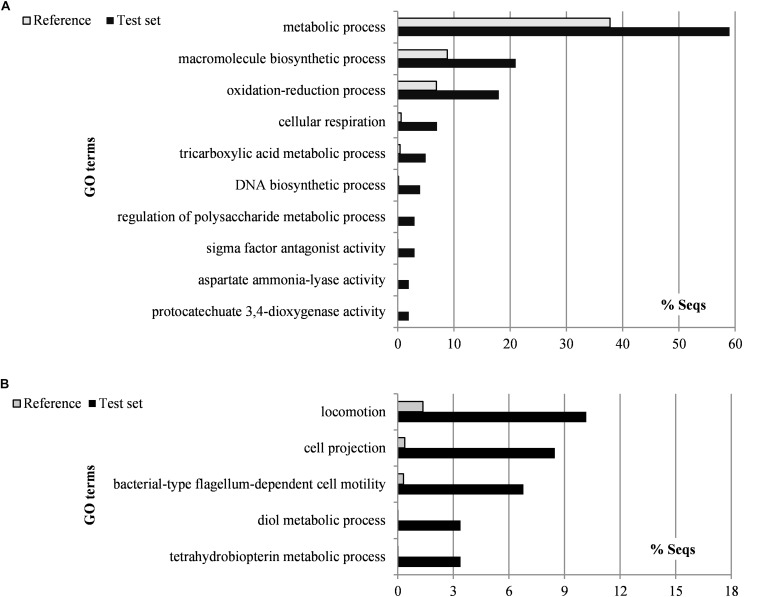
Gunpowder green tea affects the expression of *P. syringae* pv. *actinidiae* virulence-related genes. Gene Ontology categories overrepresented in differentially expressed genes that are upregulated **(A)** or downregulated **(B)** in *P. syringae* pv. *actinidiae* (Psa) cells treated with tea (0.4 mg/ml EGCG) for 24 h. GO category enrichment was analyzed using Blast2GO (Conesa and Götz, 2008).

To determine the statistical significance of the main pathways affected by tea, we carried out an enrichment analysis of the functional categories (Figure 8 and Supplementary Figure S3). The upregulated genes were found to be enriched in functional categories related to oxidation-reduction, cellular respiration, the tricarboxylic acid cycle, sigma factor antagonism, aspartate ammonia-lyase activity, and protocatechuate 3,4-dioxygenase activity. In contrast, the downregulated genes were mainly enriched in categories related to cellular motility (locomotion, cell projection, bacterial-type flagellum-dependent cell motility), as well as tetrahydrobiopterin and diol metabolism. Although functional categories related to iron metabolism were not significantly enriched, 16 genes related to pyoverdine biosynthesis and iron uptake were identified among the 159 differentially expressed genes (Table 2).

**TABLE 2:** Differentially expressed genes related to iron uptake modulated by tea treatment in *P. syringae* pv. *actinidiae*.

**Gene ID**	**Log_2_ FC**	**Adjusted *p*-value**	**Gene product description**
**Iron uptake**			
WP_004397024.1	–1.41	0.00161	Bacterioferritin
WP_002554851.1	–1.27	0.01012	Bacterioferritin
WP_003383208.1	0.52	0.06847	Pyoverdine biosynthesis regulatory protein
WP_019716573.1	0.56	0.06671	Pyoverdine side chain peptide synthetase
WP_003380601.1	0.54	0.04754	Iron-sulfur cluster transcriptional regulator
WP_017684003.1	0.67	0.02715	Chromophore maturation protein (*pvdO*)
WP_003383209.1	0.87	0.01055	Iron-regulated peptidase
WP_032701228.1	0.93	0.01807	Pyoverdine side chain peptide synthetase
WP_032701228.1	1.01	0.00375	Pyoverdine side chain peptide synthetase
WP_003382993.1	1.02	0.0124	Pyoverdine synthetase, thioesterase component
WP_003377968.1	1.13	0.01738	TonB-system energizer (*exbB*)
WP_003380249.1	1.14	0.00424	Iron ABC transporter substrate-binding protein
WP_017684367.1	1.31	0.00206	Iron-regulated peptidase
WP_003377967.1	1.33	0.00068	Biopolymer transporter (*exbD*)
WP_004396478.1	1.69	0.00733	Bacterioferritin-associated ferredoxin;
WP_017683839.1	1.87	0.00068	Fe(III) dicitrate transporter (*fecA*)

### Gunpowder Green Tea Has a Moderate Protective Effect Against Psa in Kiwifruit Plants

We carried out a preliminary experiment to investigate the protective effect of Gunpowder green tea in kiwifruit plants exposed to Psa. The plants were sprayed with tea at the half-MBC (4.7 mg/ml EGCG) 1 day before artificial infection with Psa. The preventive treatment and the infection procedure were chosen to match natural infection conditions as closely as possible, i.e., the bacterial inoculum and protectant were presented at different times rather than simultaneously.

**FIGURE 9:**
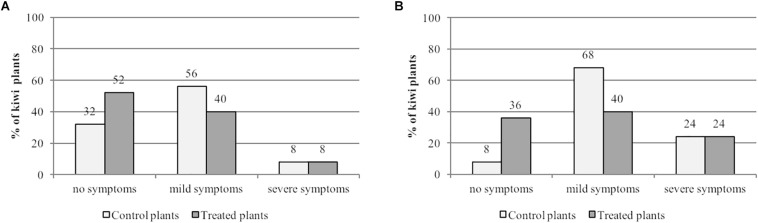
Gunpowder green tea reduces the infection of kiwi plants by P. *syringae* pv. *actinidiae*. Two-month-old *Actinidia deliciosa* cv. Hayward kiwi plants were sprayed with Gunpowder green tea (4.7 mg/ml EGCG). After 24 h, the treated and untreated (control) plants were infected with *P. syringae* pv. *actinidiae* (1 × 10 7 cells/ml). Symptoms were observed after 7 **(A)** and 14 **(B)** days. No symptoms, no necrotic spots on leaves; mild symptoms, fewer than 10 necrotic spots on apical expanding leaves; severe symptoms, more than 10 necrotic spots on apical expanding leaves.

We found that spraying with the tea significantly reduced the proportion of plants showing foliar symptoms, scored at 7 and 14 dpi (Figure 9). After 7 days, 52% of the treated plants showed no evidence of necrotic spots on expanding leaves, compared to 32% in the untreated controls (Figure 9A). Similarly, the proportion of plants displaying mild symptoms was lower in the treatment group (40%) compared to the untreated controls (56%). However, the proportion of plants showing severe symptoms was identical (8%) in both the treatment group and the untreated controls. The same general profile was observed 14 dpi (Figure 9B), i.e., a higher proportion of asymptomatic plants in the treatment group (36%) compared to the untreated controls (8%), 40% of treated plants presenting mild symptoms compared to 68% of untreated controls, and severe symptoms observed in 24% of the plants in both groups.

Given the virulence of the Psa strain CRAFRU 8.43, the first leaf spots were observed 4 dpi in the untreated plants and the number of symptomatic plants was close to 100% at 14 dpi. Treated plants were instead protected from infection, probably due to the inhibition of Psa epiphytic multiplication or motility, and started showing the first symptoms later (7 dpi) and never reached the same level of infection as the control group, even at 14 dpi.

## Discussion

Green tea is one of the most popular beverages worldwide due to its health-promoting properties, which reflect the presence of antioxidants and also antimicrobial substances that are active against various Gram-positive and Gram-negative bacteria (Lee et al., 2003; Kim et al., 2004; Taguri et al., 2004; Tiwari et al., 2005; Lo and Zhao, 2015). Both activities are mainly due to the presence of polyphenols, the most abundant of which are the catechins, particularly epigallocatechin gallate (EGCG), which represents 50–80% of the total catechin content (Khan and Mukhtar, 2007). The reported antimicrobial activity of EGCG against human pathogens (Osterburg et al., 2009; Gordon and Wareham, 2010; Liu et al., 2017) may involve the direct disruption of bacterial membrane integrity (Lee et al., 2009), as well as indirect effects such as the chelation of iron (Hider et al., 2001), the inhibition of folate biosynthesis (Navarro-Martínez et al., 2005) and the inhibition of virulence functions such as type III secretion systems or quorum sensing (Spina et al., 2008; Yin et al., 2015; Tsou et al., 2017). In particular, interference with quorum sensing may affect density-dependent functions including bacterial motility and biofilm formation (Lee et al., 2009), as well as the secretion of virulence-related enzymes such as lipases, proteases, elastases, and toxic metabolites such as pyocyanins (Loh et al., 2002). Green tea may therefore offer a new source of antimicrobial compounds that can be used to reduce our reliance on antibiotics, by inhibiting bacterial virulence without selecting for resistant bacterial strains (Otto, 2004; Truchado et al., 2009; Daglia, 2012).

The effect of green tea on human pathogens is well documented, but little is known about its effect on phytopathogenic bacteria and the antibacterial mechanism is unclear (Kodama et al., 1991; Lo and Zhao, 2015; Biancalani et al., 2016; Yang and Zhang, 2019). Green tea polyphenols can inhibit type III pilus assembly and quorum sensing genes in the olive knot pathogen *Pseudomonas savastanoi* pv. *nerii* (Biancalani et al., 2016). However, *Pseudomonas syringae* pv. *actinidiae* (Psa) may differ from other *Pseudomonas* pathogens in terms of signaling mechanisms because it lacks a canonical quorum sensing system (Patel et al., 2014). We found that Gunpowder green tea significantly inhibited the *in vitro* growth of Psa in both disk diffusion and broth microdilution assays, similar to the effect of green tea against *Staphylococcus aureus* and *Pseudomonas aeruginosa*, with MIC values similar to other strong growth inhibitors (Traczewski and Brown, 2008; Rusenova and Parvanov, 2009; Radji et al., 2013). The MIC and MBC values of Gunpowder green tea extracts were equivalent to 1 and 9.5 mg/ml EGCG, respectively. Compounds with a MBC greater than four times the MIC are usually not considered bactericidal (French, 2006).

In addition to its bacteriostatic effect, sub-MIC concentrations of Gunpowder green tea extract (here defined as 0.4 mg/ml EGCG) influenced other important virulence traits in Psa, including swimming motility and biofilm formation, and to a lesser extent iron chelation. Green tea has previously been shown to have similar effects against *Escherichia coli*, *Pseudomonas putida*, *Burkholderia cepacia* and *Dictyostelium discoideum* at sub-MIC concentrations of EGCG (Huber et al., 2003; Lee et al., 2009; McQuade et al., 2013). We found that the inhibition of Psa swimming motility by tea extracts fitted well with the downregulation of genes responsible for motility and flagellum-related functions (*fleQ*, *fliT* and *flagellin*), prompting us to examine the molecular basis of this phenomenon in more detail by microarray analysis. The inhibition of these genes is also associated with the downregulation of transcriptional regulators of the GntR and MerR families, both of which play a positive role in motility (Yeung et al., 2009; Su et al., 2016). The disruption of Psa motility pathways by green tea may in part explain the lower ability of Psa to colonize kiwifruit plants after spraying. Indeed, flagellum-dependent motility is essential for the colonization of plant tissues. In particular, flagella are required to make initial adhesive contacts with the plant (Lawrence et al., 1987) and/or to bring the bacterial cell into close proximity with the surface (Mills and Powelson, 1996).

Unlike the motility-dependent virulence functions described above, biofilm formation is a colony non-motile condition, in which bacteria attach to a surface and reside within a matrix composed of exopolysaccharides, proteins and DNA (Flemming and Wingender, 2010). This matrix protects bacteria from external insults, including osmotic and oxidative stress and antibiotics (Wai et al., 1998; Bogino et al., 2013). Accordingly, the upregulation of genes related to biofilm synthesis in the presence of tea may indicate that Psa attempts to protect itself against the antimicrobial activity of the extract, although other hypotheses may explain this behavior. However, gene expression analysis did not reveal a quantitative correlation with biofilm synthesis: sub-MIC concentrations of tea did not promote biofilm formation, and even prevented the formation of a weak Psa biofilm under normal growth conditions. One potential explanation is that the inhibition of bacterial motility may account for the anti-biofilm activity of the tea, given that bacterial motility is required for cells to reach the attachment surface in the first stage of biofilm formation (O’Toole and Kolter, 1998). Accordingly, a non-motile strain of *Agrobacterium tumefaciens* was reported to be unable to produce a biofilm (Merritt et al., 2007). Interestingly, higher concentrations of tea promoted the formation of biofilms *in vitro*, perhaps because such concentrations represent highly unfavorable growth conditions for Psa, leading to a low metabolic activity and thus triggering aggregation behavior as an ultimate survival strategy (Bogino et al., 2013). Furthermore, such high concentrations of green tea extract may provide Psa with large quantities of plant polysaccharides as a matrix to promote initial attachment and colony formation (Beauregard et al., 2013). Up to 4% of the dry mass of green tea is carbohydrate, and extracts were shown to promote biofilm formation by *P. syringae* pv. *theae* even at low sub-inhibitory concentrations of EGCG, probably via cyclic diguanylate signal transduction (Horie and Kobata, 2002; Tomihama et al., 2007). *P. syringae* pv. *theae* is the closest known relative of Psa (Bull et al., 2011), so both strains may share sensors and/or signaling pathways allowing a similar mechanism to occur in Psa. The reduction of motility in the presence of tea could at the same time prevent biofilm dispersion, which relies on bacterial movement for structural disassembly and release from the biofilm matrix (Klausen et al., 2003). Hence, high concentrations of sugars and limited biofilm dispersion could ultimately contribute to biofilm development under such conditions.

Another important bacterial virulence trait (in epiphytic and endophytic lifestyles) is the ability to chelate iron by secreting siderophores (Lamont et al., 2002). Most polyphenolic compounds in plant extracts are known to chelate metals and could therefore play a role in iron starvation, thus reducing bacterial fitness (Hider et al., 2001; Thode et al., 2015; Lagha et al., 2017). Accordingly, green tea polyphenols chelate iron to form iron(III) complexes with stability constants comparable to iron-siderophore complexes (Hider et al., 2001; Elhabiri et al., 2007). In our experiments, the presence of Gunpowder green tea extracts correlated with slightly reduced bacterial siderophore activity, although the effect was probably underestimated due to the above-mentioned competitive iron-binding activity exerted by green tea itself. Surprisingly, Psa genes involved in siderophore synthesis (*pvdE*, *pvdS*, and *pvdO*) were upregulated in the presence of tea, thus showing an inverse correlation with the reduction in iron chelation. Nutrients and phenolic compounds in leaf exudates can induce siderophore production because they sequester iron (Karamanoli and Lindow, 2006). The induction of Psa genes that promote iron uptake by tea may therefore represent a compensatory mechanism allowing the bacteria to cope with iron starvation, as already reported in *P. fluorescens* treated with EGCG (Liu et al., 2017). Moreover, green tea polyphenols (including EGCG) induce oxidative stress and DNA damage by releasing H_2_O_2_ (Cui et al., 2012; Liu et al., 2013, 2017). In particular, *P. fluorescens* cells exposed to EGCG produce H_2_O_2_ that oxidizes and thus inactivates the transcriptional repressor Fur (which regulates iron uptake), resulting in the induction of genes responsible for iron acquisition (Varghese et al., 2007; Liu et al., 2017). The upregulation of GO functional categories related to oxidation-reduction processes indicates the oxidative stress caused by the tea extract may contribute to the induction of iron uptake mechanisms associated with the perception of iron starvation.

Despite the large body of evidence concerning the antimicrobial activity of green tea, few studies have addressed the transcriptome-wide impact of polyphenols in bacteria to explain how compounds such as EGCG affect bacterial metabolism and signaling (Carraro et al., 2014; Liu et al., 2017). However, this information is needed for the rational development of sustainable strategies to control plant diseases. As stated above, Gunpowder green tea extract cause extensive transcriptomic reprogramming in Psa, not only shedding light on the molecular basis of virulence but also highlighting groups of upregulated genes belonging to functional categories such as macromolecule biosynthesis, DNA biosynthesis, and metabolic processes. The latter included the tricarboxylic acid cycle, an important aerobic metabolic pathway responsible for the production of ATP by the generation of reducing molecules (NADH and FADH_2_) required for bacterial survival (Resch et al., 2005). These data strongly suggest that Psa remains metabolically active in the presence of sub-MIC concentrations of tea (0.4 mg/ml EGCG) thus confirming its bacteriostatic and anti-virulence activity. Together these data demonstrate not only that green tea is an efficient anti-bacterial agent that acts against the phytopathogen Psa by reducing its virulence and fitness *in planta*, but also that neither metabolism nor viability are affected at the molecular level. This is necessary for the development of sustainable control strategies that prevent the emergence of new resistant strains.

## Data Availability Statement

The custom design of microarray chip can be found in the GEO platform under the accession number GPL27505 (https://www.ncbi.nlm.nih.gov/geo/query/acc.cgi?acc=GPL27505). The datasets of gene expression generated by microarray for this study have been deposited in NCBI GEO repository under the series record GSE137806 (https://www.ncbi.nlm.nih.gov/geo/query/acc.cgi?acc=GSE137806).

## Author Contributions

AL performed all the experiments and wrote the manuscript. APi participated in the experiments on the phenotypic effects of tea treatments. NV performed microarray data analyses. APo and EV conceived the study, supervised the experiments, and wrote the manuscript.

## Conflict of Interest

The authors declare that the research was conducted in the absence of any commercial or financial relationships that could be construed as a potential conflict of interest.

## Footnotes

1 http://www.pseudomonas.com/strain/show/521

2 https://earray.chem.agilent.com/earray/

3 https://www.ncbi.nlm.nih.gov/geo/query/acc.cgi?acc=GPL27505
