# Supplementary material for: Inhibition of Virulence-Related Traits in Pseudomonas syringae pv. actinidiae by Gunpowder Green Tea Extracts
Source: Front Microbiol. 2019 Oct 11;10:2362. doi: 10.3389/fmicb.2019.02362 (PMC6797950; doi:10.3389/fmicb.2019.02362)
Supplement: Supplementary file 1 [file Data_Sheet_1.PDF]

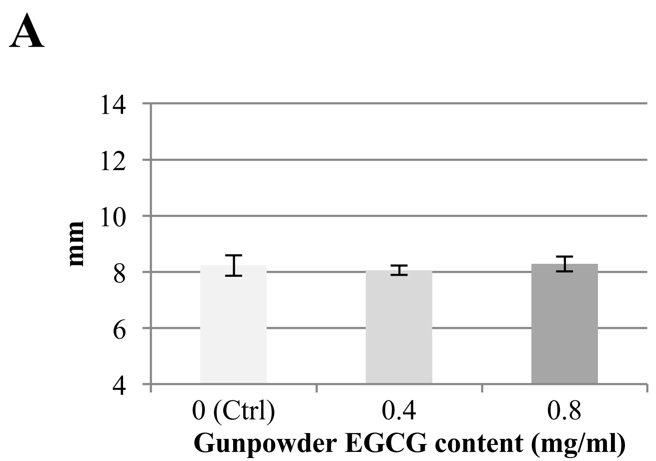

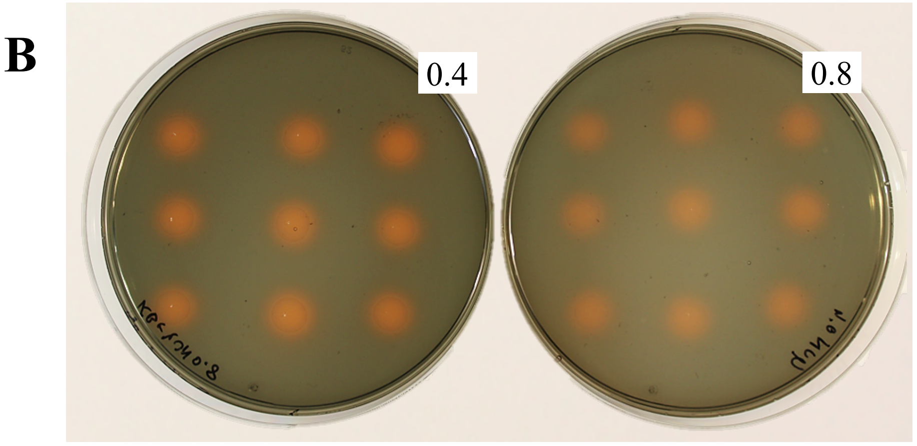

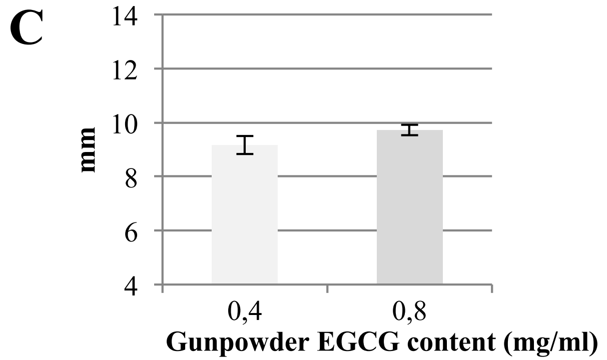

Supplementary Figure S1. We cultivated *P. syringae* pv. *actinidiae* for 24 h in KB medium (Ctrl) or KB medium with tea (0.4 and 0.8 mg/ml EGCG), spotted 12.5 µl of the culture onto CAS agar plates and incubated them for 24 h at 28°C. (**A**) Colony diameters measured after 24 h. (**B**) Tea (0.4 and 0.8 mg/ml EGCG) was spotted alone onto CAS agar plates, producing orange haloes due to the iron chelating ability of green tea polyphenols. (**C**) Size of the haloes after 1 h. Values are expressed as means ± SD of triplicate assays from three independent experiments. Asterisks indicate a statistically significant difference (**p < 0.01) according to Student's t-test.

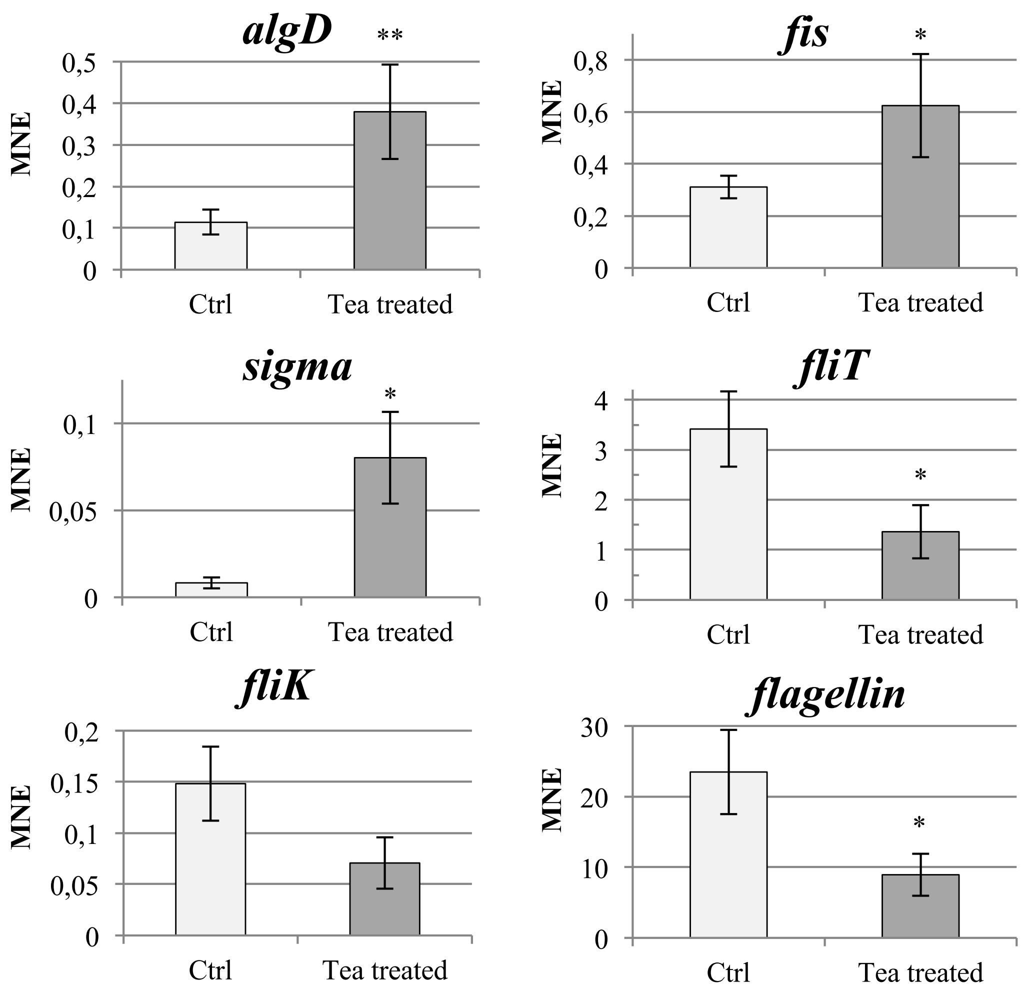

Supplementary Figure S2. Gunpowder green tea modulates the expression of genes with diverse functions in *P. syringae pv. actinidiae*. Expression profiles of genes encoding GDP-mannose 6-dehydrogenase (*algD*; WP_017683639.1), *fis* family transcriptional regulator (*fis*; WP_002555375.1), RNA polymerase sigma factor (*sigma*; WP_005737468.1), flagellar assembly protein (*fliT*; WP_002554297.1), flagellar hook-length control protein (*fliK*; WP_017683767.1) and flagellin (*flagellin*; WP_003382135.1) in tea tea-treated *P. syringaepv. actinidiae* (Psa) cells (cultured for 24 h in the presence of tea, 0.4 mg/ml EGCG) compared to untreated (Ctrl) cells cultured in KB medium without tea. The expression levels were determined by qRT-PCR and normalized to *rpoD* expression (WP_017683803.1). Data are expressed as mean expression values (MNEs) ± SD of three independent experiments. Asterisks represent statistically significant differences between tea-treated and control samples (**$p < 0.01$; *$p < 0.05$) according to Student's t-test.

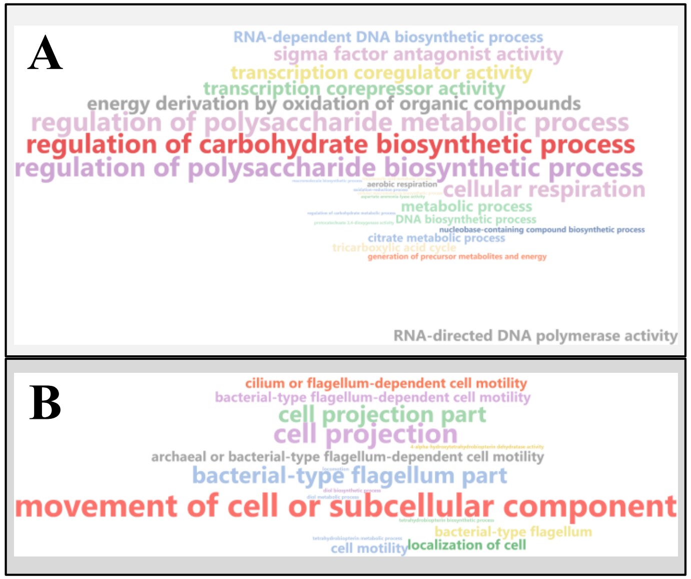

Supplementary Figure S3. Gunpowder tea modulates Psa gene categories related to virulence. Gene Ontology categories overrepresented in differentially expressed genes that are upregulated (**A**) or downregulated (**B**) in Psa cells treated with tea (0.4 mg/ml EGCG) for 24 h. GO category enrichment was analyzed using Blast2GO (Conesa et al., 2008) and is shown as a word-cloud graphical representation.
