# Supplementary material for: Inhibition of Virulence-Related Traits in Pseudomonas syringae pv. actinidiae by Gunpowder Green Tea Extracts
Source: Front Microbiol. 2019 Oct 11;10:2362. doi: 10.3389/fmicb.2019.02362 (PMC6797950; doi:10.3389/fmicb.2019.02362)
Supplement: Supplementary file 2 [file Table_1.docx]

**Supplementary Table S1.** List of primer sequences used for quantitative RT-PCR.

| **Target gene and product** | **Protein ID** | **Primer** | **Primer sequence**  **(5’→3’)** |
| --- | --- | --- | --- |
| ***algU***  (RNA polymerase  sigma factor) | WP_003378493.1 | AlgU.For | TTACGAAGACATTGCGAGCGT |
|  |  | AlgU.Rev | CAACGGCTGCAGGGCTTTAT |
| ***aprA***  (serine 3-  dehydrogenase) | WP_017682688.1 | AprA.For | GATTCCAACCGTGCCGCT |
|  |  | AprA.Rev | GCTGCTCTGGTTGTAGTTC |
| ***fleQ***  (ATPase AAA) | WP_003382141.1 | FleQ.For | GAGCGTGTCGGCAGCAAC |
|  |  | FleQ.Rev | AGGGAACACATTGAGACGG |
| ***gacA***  (chemotaxis  protein CheY) | WP_005737926.1 | GacA.For | CGGAACGCCAGAATGAATC |
|  |  | GacA.Rev | CAGTGAGGCGAGCAGCAT |
| ***hrpC***  (hrcJ family type III  secretion inner membrane  ring protein) | WP_003375729.1 | HrpC.For | TCCAGACGTTTACGCGCATC |
|  |  | HrpC.Rev | GGCTGCAGTGATGAGACTGA |
| ***hrpW1***  (type III helper  protein) | WP_017682518.1 | HrpW1.For | GGCTGCAGTGATGAGACTGA |
|  |  | HrpW1.Rev | TCGTTCTCACCCAGGTTCAC |
| ***Lip_1***  (annotated lipase) | WP_025987999.1 | Lip_1.For | ATTATCTGAGCGACCGTGC |
|  |  | Lip_1.Rev | CGGGTAGGAAGACTCACGG |
| ***pilM***  (pilus assembly  protein) | WP_003378852.1 | PilM.For | CGATTATGTCAGCGAAGTG |
|  |  | PilM.Rev | CCTGAAATCGAAGCCGTAC |
| ***pvdE***  (cyclic peptide  transporter) | WP_017684002.1 | PvdE.For | CGGGTTGTGTGTGCTGAC |
|  |  | PvdE.Rev | ACGGTGCGAGCGGTAGCG |
| ***pvdO***  (chromophore  maturation  protein) | WP_017684003.1 | PvdO.For | CGGAAATGGTCGTGCTGCC |
|  |  | PvdO.Rev | TACCTGAAACTGGCTGATGG |
| ***pvdS***  (pyoverdine sidechain  peptide synthetase IV) | WP_019716667.1 | PvdS.For | GTGATGCCGCTGACCGCC |
|  |  | PvdS.Rev | GTTCAGACCGACCCGCTC |
| ***rpoD***  (RNA polymerase  sigma factor) | WP_017683803.1 | RpoD.For | GTTGCCCTTGCCGAATTGTT |
|  |  | RpoD.Rev | CATCACGTACGCACAACTGC |
| ***algD***  (GDP-mannose 6-dehydrogenase) | WP_017683639.1 | AlgD.For | GCCAACAAGGACTACATCG |
|  |  | AlgD.Rev | GTCTTGTTGGCGAGGGCG |
| ***fis***  (*fis* family transcriptional regulator) | WP_002555375.1 | Fis.For | CTGGTGTTGTCGGAAGTCG |
|  |  | Fis.Rev | GCGATTGAGCCCCAGCAG |
| ***sigma***  (RNA polymerase  sigma factor) | WP_005737468.1 | Sigma.For | CGAAGCCGAGCAGCCGTC |
|  |  | Sigma.Rev | ATAGAGAAACGCAGCCCGC |
| ***flagellin*** | WP_003382135.1 | Flagellin.For | TGGGTGTCGGTTCGGCAG |
|  |  | Flagellin.Rev | GGTGGAGTTGATGGTCTGC |
| ***fliK***  (flagellar hook-length control protein) | WP_017683767.1 | Flik.For | TGGAGCAGACCCGCACCC |
|  |  | Flik.Rev | GCGGTCTTCGGTGGTTTCC |
| ***fliT***  (flagellar assembly protein) | WP_002554297.1 | FliT.For | CCTTGGTCGGGGCGTTGG |
|  |  | FliT.Rev | CAGGTTCGTCGGCAGGCG |
